# Impaired glucose regulation, depressive symptoms, and health-related quality of life

**DOI:** 10.1136/bmjdrc-2020-001568

**Published:** 2020-10-19

**Authors:** Jannica S Selenius, Niko S Wasenius, Hannu Kautiainen, Minna Salonen, Mikaela von Bonsdorff, Johan G Eriksson

**Affiliations:** 1 Department of General Practice and Primary Health Care, University of Helsinki, Helsinki, Finland; 2 Public Health Research Porgramme, Folkhälsan Research Center, Helsinki, Finland; 3 Department of Epidemiology and Health Promotion, National Public Health Institute, Helsinki, Finland; 4 Gerontology Research Center and Faculty of Sport and Health Sciences, University of Jyväskylä, Jyväskylä, Finland; 5 Singapore Institute for Clinical Sciences (SICS), Agency for Science, Technology and Research (A*STAR), Singapore; 6 Department of Obstetrics & Gynecology, Yong Loo Lin School of Medicine, National University of Singapore, Singapore

**Keywords:** diabetes mellitus, type 2, insulin resistance, quality of life, depression

## Abstract

**Introduction:**

This study aims to investigate whether the associations between impaired glucose regulation and health-related quality of life are modified by severity or type of depressive symptoms.

**Research design and methods:**

For this cross-sectional study, we included 1939 individuals (mean age 61.5 years) from the Helsinki Birth Cohort Study. Between 2001 and 2004, a standard 2-hour 75 g oral glucose tolerance test was applied to define normoglycemia, pre-diabetes, and newly diagnosed diabetes. Information on previously diagnosed diabetes was collected from national registers and questionnaires. Pre-diabetes was defined as having either impaired fasting glucose or impaired glucose tolerance. The Mental and Physical Component Scores of health-related quality of life were assessed with Short Form-36. Beck’s Depression Inventory was employed to investigate the severity of depressive symptoms and to define minimal (depression score <10), non-melancholic, and melancholic types of depression. We analyzed data with general linear models adjusted for sex, age, lifestyle factors, comorbidities, and body mass index.

**Results:**

Glucose regulation subgroups, especially previously known diabetes, were associated with lower Physical Component Score (p=0.001) and higher depression score (p=0.015), but not with the Mental Component Score (p=0.189). Non-melancholic depression was associated with lower Physical and Mental Component Scores compared with those with depression score <10 and melancholic depression (p<0.001), independently of glucose regulation status (p for glucose regulation status by depression type interaction >0.54).

**Conclusions:**

Non-melancholic type of depression and previously known diabetes are independently associated with lower health-related quality of life. This should be appraised in long-term treatment of diabetes and when treating non-melancholic depressive symptoms to maintain a higher health-related quality of life.

**Significance of this study**

**What is already known about this subject?**

- Diabetes and depression are widespread diseases on the rise with a considerable negative influence on health-related quality of life.

**What are the new findings?**

- In addition to contributing to the perception that previously known diabetes is associated with poorer health-related quality of life, we found that decreased health-related quality of life is seen in subjects with non-melancholic depressive symptoms regardless of glucose regulation status.

**How might these results change the focus of research or clinical practice?**

- These results highlight the importance of maintaining mental health in individuals suffering from diabetes to prevent health-related quality of life from deteriorating, further our findings promote more research aiming at characterizing type of depressive symptoms associated with impairment in glucose regulation.

## Introduction

Globally, both type 2 diabetes and depression show a high and increasing prevalence. Diabetes is estimated to affect 463 million people globally,^1^ the corresponding number for depression being over 300 million.^2^ Diabetes attributed to 5 million deaths in 2017 while depression was the leading cause for suicide globally.^2 3^ In addition, being global health burdens, diabetes and depression affect the individuals suffering from the disorders in several ways, with impact on physical and mental health and overall well-being.^4–6^

Diabetes is strongly associated with both depression and poorer quality of life.^7^ Poorer quality of life and non-optimal mental health can influence diabetes care negatively, predisposing to worse glycemic control and an increased risk for diabetes-associated complications.^4^ According to a recent meta-analysis, individuals with diabetes were around 30% more likely to develop depression than individuals with normoglycemia (NGT).^8^ Some studies have reported no association between pre-diabetic and depressive symptoms, suggesting that depression stems more from the burden of diabetes than from insulin resistance, which is a characteristic feature of the pre-diabetic states.^9 10^ Furthermore, compared with newly diagnosed diabetes, previously diagnosed diabetes has been more closely linked to both depression and poorer quality of life.^8^

Metabolic syndrome is characterized by disturbances in glucose and lipid metabolism as well as adiposity and is a major risk factor for the development of type 2 diabetes. Studies have reported a linkage between metabolic syndrome and subgroups of depression, that is, mainly for non-melancholic and melancholic depression, and metabolic syndrome.^11–13^ Non-melancholic depression is characterized by hypersomnia and weight gain, whereas melancholic depression is linked with loss of appetite and disturbances in affect and psychomotor functions.^14^ Non-melancholic depression has been proposed to show a stronger association with inflammatory diseases, while melancholic depression mainly is a disease of the central nervous system not associated with systemic processes.^14 15^

Although the link between type 2 diabetes and depression has been studied, less is known about the type of depression associated with different degrees of impairment in glucose regulation. Furthermore, only few studies have focused on the relationship between pre-diabetes and health-related quality of life (HRQoL). Previous studies have reported that previously known diabetes is associated with poorer physical and mental components of HRQoL.^4 5 16^ Only a few studies have investigated the relationship between pre-diabetes and HRQoL, and the findings have been inconsistent.^17–19^

The aim of this study was to investigate whether the association between the different degrees of impairment in glucose regulation and HRQoL is modified by the severity and type of depressive symptoms.

## Research design and methods

### Participants

**Figure 1:**
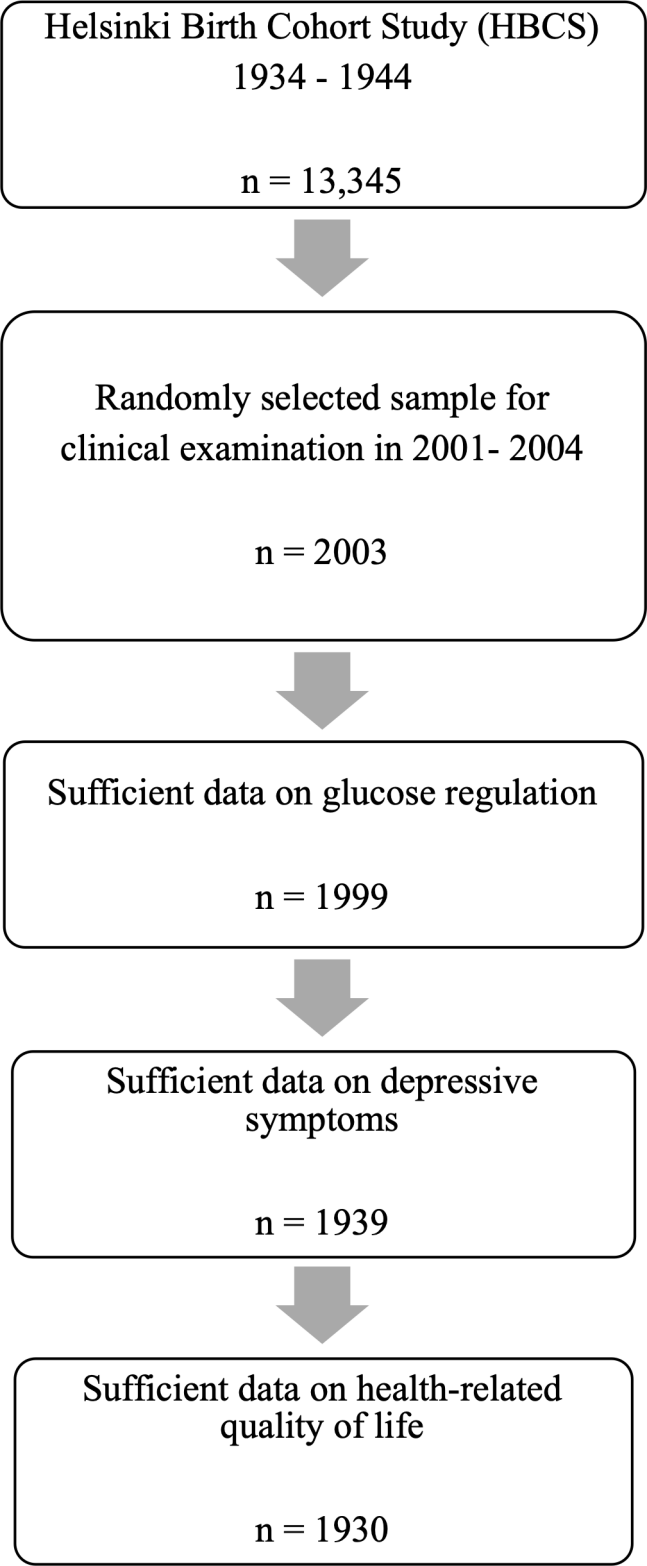
Flow chart of the data screening process.

The Helsinki Birth Cohort Study (HBCS) includes 13 345 individuals born between 1934 and 1944 at the Helsinki University Central Hospital or the Helsinki City Maternity Hospital.^20^ They attended child welfare clinics in Helsinki and were living in Finland in 1971 when a unique personal identification number was allocated to all individuals of the Finnish population.^21^ A baseline clinical examination was conducted between 2001 and 2004, involving 2003 cohort members. Of those, 1999 had sufficient data on glucose regulation status and were included in this study. After excluding individuals with missing information, 1939 had sufficient information on depressive symptoms and 1930 on HRQoL (figure 1).

### Glucose regulation

The WHO 2006 criteria^22^ were used for diagnosing diabetes, impaired glucose tolerance (IGT), and impaired fasting glucose (IFG). Fasting plasma glucose was measured in all individuals participating in the clinical examination and a standard 2-hour 75 g oral glucose tolerance test (OGTT) was conducted, except for those with previously known diabetes. Previously known diabetes was defined by self-report, register linkage, or use of medication for diabetes. Individuals who received a diagnosis of diabetes for the first time based on the OGTT were classified as having newly diagnosed diabetes. Individuals who met the criteria for both IFG and IGT were classified as having IGT. IFG or IGT were grouped together and called pre-diabetes.

### Health-related quality of life

HRQoL was assessed using the validated Finnish 36-Item Short Form Health Survey (SF-36) version 1.0 questionnaire.^23^ The average age at the time of filling the questionnaire was 61.5 years (SD 2.9). The SF-36 consists of eight domains: physical functioning (10 items), role limitations caused by physical health problems (four items), role limitations caused by emotional health problems (three items), bodily pain (two items), general health (five items), vitality (four items), mental health (five items), and social functioning (two items). Possible scores for each item ranged from 0 (lowest perceived functioning) to 100 (highest perceived functioning). Physical and mental health component scores were aggregated using the US reference population (1990) to standardize the eight domains and for factor score coefficients. The summary scores were standardized using a mean of 50 and a SD of 10. The Finnish SF-36 has been reported as reliable and well validated.^24^

### Depression

Depression was screened for using the validated 21-item Beck’s Depression Inventory (BDI) questionnaire. The psychometric properties of the Finnish BDI have been well validated.^25 26^ Patients who scored 10 or higher were considered to suffer from depression, as previously suggested.^27^ Participants with a BDI score ≥10 were divided into melancholic and non-melancholic types of depression^28^ based on the Diagnostic and Statistical Manual of Mental Disorders, fourth edition criteria. Melancholic symptoms in the BDI (sadness, past failure, loss of pleasure, feelings of guilt, punishment feelings, loss of interest, irritability, change of sleeping, and appetite) were applied to divide the participants into melancholic and non-melancholic types of depression.^12 29–31^

### Covariates

At the clinical examination, subjects were asked about their current health situation, use of medication, and lifestyle characteristics. Socioeconomic status was obtained from Statistics Finland and was coded as high official, low official, self-employed, and laborer based on the original classification system.^32^ Smoking was coded as never, former, and current, and alcohol use was coded as never or having quit, less than once a week, or weekly. Anthropometrics including weight and height were measured and body mass index (BMI) was calculated as weight in kilograms divided with height in meters squared. At the clinical examination, performed by three trained study nurses, the cohort members were asked about their past 12-month leisure-time physical activity using a validated exercise questionnaire: the Kuopio Ischemic Heart Disease Risk Factor Study.^33^ Leisure-time physical activity was measured in metabolic equivalents of task (MET),^34^ which were multiplied with time (hours) and frequency to calculate MET-hours as previously suggested.^35^ Cohort members were also asked about chronic diseases and conditions, including cardiovascular conditions (congestive heart failure, arrhythmias, claudication, angina pectoris, previous heart attack, and stroke), lung diseases (asthma, emphysema, and chronic bronchitis), musculoskeletal disorders (rheumatoid arthritis, osteoporosis) and presence of cancer using validated questionnaires. The presence of comorbidities was coded as none, one, two, or more.

### Statistical analysis

Characteristics of the cohort members were compared across glucose regulation groups using general linear models. In the case of violation (eg, non-normal distribution), a bootstrap-type analysis with 2500 replications was used. General linear models were also applied to investigate the associations between glucose regulation status and physical functioning, mental functioning, and depressive symptoms. Adjustments were first made for sex and age, then for lifestyle factors (smoking status, alcohol consumption, socioeconomic status, leisure-time physical activity, and comorbidities), and lastly for BMI as our early models showed a relatively strong effect by BMI when controlling for it. All tests were performed two-tailed and the level of significance was set at p<0.05. Statistical analyses were carried out using Stata Version/MP V.15.1 (Stata, College Station, Texas, USA) and IBM SPSS Statistics V.25 (IBM, Armonk, New York, USA).

## Results

### Participants’ characteristics

This study included 897 men and 1042 women. The mean age at the time of the clinical examination was 61.5 years for both sexes (SD 2.8 for men and 3.0 for women). The characteristics of the cohort members across the glucose regulation groups—that is, NGT, pre-diabetes, newly diagnosed diabetes, and previously known diabetes—are shown in table 1.

**Table 1:** Characteristics of the study population according to glucose regulation

Characteristics	Glucose regulation status				P value
	NGT (N=1010)	Pre-diabetes (N=610)	Newly diagnosed diabetes (N=181)	Previously known diabetes (N=134)	
Sex, n (%)					<0.001
Male	427 (42)	293 (48)	103 (57)	73 (54)	
Female	578 (58)	317 (52)	78 (43)	61 (46)	
Age, years	61.3 (2.8)	61.9 (3.1)	61.8 (2.8)	61.4 (3.0)	0.001
BMI, kg/m^2^	26.4 (4.1)	28.0 (4.3)	29.9 (5.1)	30.9 (5.6)	<0.001
Smoking, n (%)					0.015
Current	239 (24)	134 (22)	50 (28)	30 (22)	
Quit	322 (32)	210 (34)	68 (38)	61 (46)	
Never	444 (44)	266 (44)	63 (35)	43 (32)	
Alcohol use, n (%)					0.002
Weekly	516 (51)	345 (57)	84 (46)	51 (38)	
Less than weekly	411 (41)	230 (38)	80 (44)	74 (55)	
Never/quit	78 (8)	35 (6)	17 (9)	9 (7)	
Socioeconomic status, n (%)					0.069
High official	162 (16)	82 (13)	18 (10)	17 (13)	
Low official	436 (43)	275 (45)	72 (40)	47 (35)	
Self-employed	82 (8)	59 (10)	20 (11)	16 (12)	
Laborers	325 (32)	194 (32)	71 (39)	54 (40)	
LTPA MET-hour/week, median (IQR)	39.5 (42.0)	33.7 (39.5)	30.7 (36.8)	30.7 (37.6)	0.020
Comorbidities, n (%)					
None	699 (70)	424 (70)	113 (62)	80 (60)	<0.001
One	235 (23)	132 (22)	37 (20)	25 (19)	
Two or more	71 (7.0)	54 (9)	31 (17)	29 (22)	

Pre-diabetes = impaired fasting glucose and impaired glucose tolerance.

Newly diagnosed diabetes = diabetes diagnosed with OGTT at clinical examination in 2001–2004.

Previously known diabetes = diabetes diagnosed before the clinical examination in 2001–2004.

1 MET = 3.5 mL of O_2_/kg/min.

BMI, body mass index; LTPA, leisure time physical activity; MET-h, metabolic equivalent of task per hour; NGT, normal glucose tolerance; OGTT, oral glucose tolerance test.

### Subgroups of glucose regulation and HRQoL and BDI

As described in table 2, in the HRQoL subscales, previously known diabetes was associated with lower physical functioning, general health, vitality, social functioning, and PCS after the model was fully adjusted for covariates. In addition, we detected a significant (p<0.02) association between the physical and emotional role limitations and bodily pain after adjusting for sex, age, smoking status, alcohol consumption, socioeconomic status, leisure-time physical activity, and comorbidities in patients with previously known diabetes. However, these associations attenuated after further adjusting for BMI (p>0.073). Previously known diabetes was also associated with higher BDI score (table 2).

**Table 2:** Association between the subgroups of glucose regulation and HRQoL and depression scores

Variables	NGT (n=1005)	Pre-diabetes (n=610)	Newly diagnosed diabetes (n=181)	Previously known diabetes (n=134)	P-values for between-group comparison		
					Model 1	Model 2	Model 3
HRQoL							
Physical functioning	85.0 (16.8)	82.3 (18.2)	80.1 (17.5)	71.4 (23.1)	<0.001	<0.001	<0.001
General health	66.4 (18.1)	63.8 (17.9)	60.1 (18.5)	51.7 (17.8)	<0.001	<0.001	<0.001
Vitality	71.8 (18.9)	70.4 (19.2)	70.3 (20.8)	64.4 (20.7)	<0.001	0.001	0.012
Mental health	81.9 (14.4)	80.6 (15.0)	81.6 (15.7)	79.8 (17.2)	0.086	0.099	0.160
Physical role limitations	82.6 (30.6)	80.7 (33.0)	78.3 (34.6)	72.2 (36.4)	0.001	0.020	0.459
Emotional role limitations	85.1 (28.4)	84.2 (31.2)	81.8 (32.3)	75.4 (36.3)	0.001	0.007	0.073
Social functioning	90.2 (17.9)	90.0 (16.8)	90.3 (16.6)	83.5 (21.1)	<0.001	0.001	0.007
Bodily pain	78.6 (22.0)	78.6 (22.4)	77.9 (21.8)	70.6 (25.7)	<0.001	0.012	0.070
PCS	49.0 (8.5)	48.2 (8.7)	47.0 (8.1)	43.2 (10.0)	<0.001	<0.001	0.001
MCS	54.2 (8.7)	53.9 (8.9)	54.3 (9.0)	52.9 (10.8)	0.267	0.231	0.189
BDI	5.4 (4.9)	5.9 (5.1)	5.8 (5.9)	7.4 (5.7)	<0.001	0.001	0.015

Data are shown as mean (SD). Model 1 adjusted for age and sex. Model 2 adjusted for Model 1+smoking, alcohol usage, socioeconomic status, comorbidities, and leisure-time physical activity. Model 3 adjusted for Model 2+body mass index.

Pre-diabetes = impaired fasting glucose and impaired glucose tolerance,

Newly diagnosed diabetes = diabetes diagnosed with the OGTT at clinical examination in 2001-2004; Previously known diabetes = diabetes diagnosed before the clinical examination in 2001–2004.

BDI, Beck’s Depression Inventory; HRQoL, health-related quality of life; MCS, Mental Component Score; NGT, normal glucose tolerance; PCS, Physical Component Score.

### HRQoL according to types of depressive symptoms across glucose regulation subgroups

**Figure 2:**
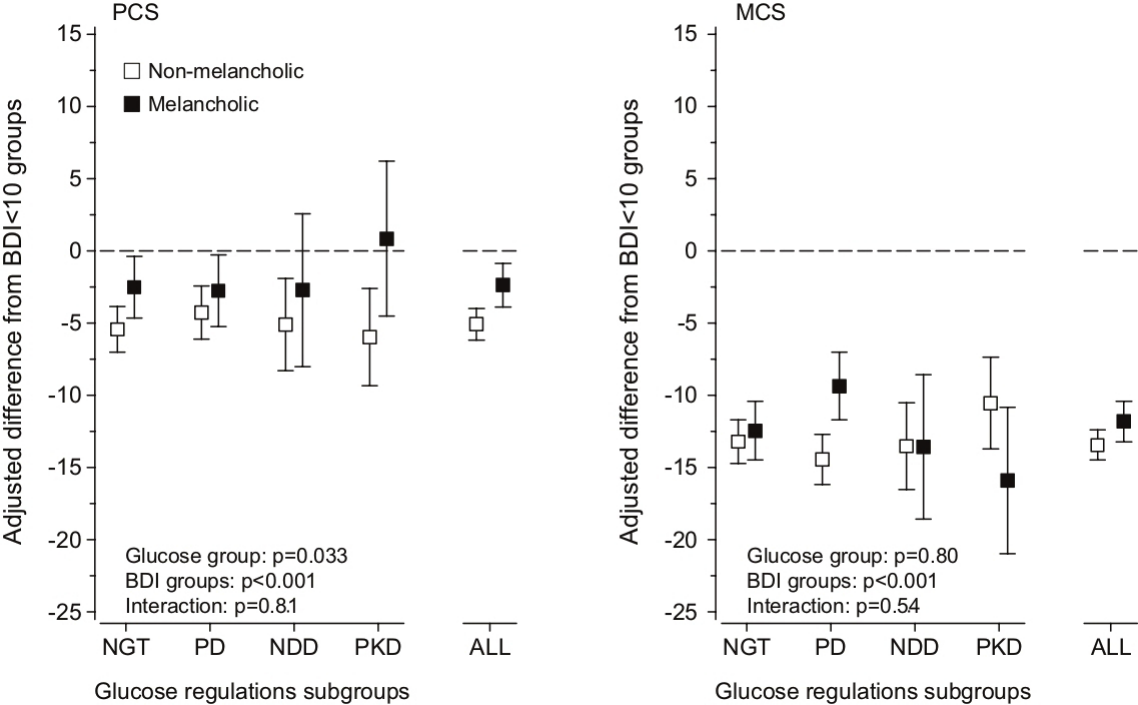
Adjusted differences of non-melancholic and melancholic depression types from the Beck’s Depression Inventory (BDI) <10 group in the Physical Component Score (PCS) and Mental Component Score (MCS) of health-related quality of life (HRQoL) among subgroups of glucose regulation. Analyses are adjusted for sex, age, smoking status, alcohol consumption, socioeconomic status, leisure-time physical activity, comorbidities, and body mass index. Error bars indicate 95% CIs.; ALL, all groups; NDD, newly diagnosed diabetes; NGT, normal glucose tolerance; PD, pre-diabetes; PKD, previously known diabetes.

Both non-melancholic and melancholic depressive symptoms were associated with lower Physical Component Score (PCS) and Mental Component Score (MCS) compared with those with a BDI score <10 regardless of glucose regulation status (table 3 and figure 2). No significant interaction was detected between the different groups of impairment in glucose regulation and type of depressive symptoms on the HRQoL components (figure 2).

**Table 3:** PCS and MCS of health-related quality of life among subjects with depressive symptoms according to glucose regulation subgroups

Variables	BDI <10	Non-melancholic depressive symptoms	Melancholic depressive symptoms	P-values for between-group comparison		
				Model 1	Model 2	Model 3
Normal glucose tolerance (n=1005)						
n	839	110	56			
PCS	50.0 (7.6)	42.8 (10.1)	46.2 (10.7)	<0.001	<0.001	<0.001
MCS	56.4 (6.1)	43.3 (11.9)	44.0 (11.1)	<0.001	<0.001	<0.001
Pre-diabetes (n=610)						
n	483	85	42			
PCS	49.4 (8.1)	43.0 (9.7)	44.9 (9)	<0.001	<0.001	<0.001
MCS	56.5 (5.7)	42.2 (11)	47.6 (11.4)	<0.001	<0.001	<0.001
Newly diagnosed diabetes (n=181)						
n	144	28	9			
PCS	48.1 (7.4)	42.0 (10)	44.2 (6.6)	0.003	0.005	0.015
MCS	57.1 (6.2)	43.5 (10.5)	43.6 (10)	<0.001	<0.001	<0.001
Previously known diabetes (n=134)						
n	98	27	9			
PCS	44.7 (9.3)	37.4 (11.2)	44.3 (9.1)	0.006	0.014	0.064
MCS	56.1 (6.6)	45.5 (14.2)	40.5 (16.2)	<0.001	<0.001	<0.001

Data are shown as mean (SD). Model 1: adjusted for age and sex. Model 2: adjusted for Model 1+smoking, alcohol usage, socioeconomic status, comorbidities and leisure-time physical activity. Model 3: adjusted for model 2+body mass index.

BDI, Beck's Depression Inventory; MCS, Mental Component Score; PCS, Physical Component Score.

## Conclusions

Our findings showed no interaction between impairment in glucose regulation and type of depressive symptoms on HRQoL. However, both types of depressive symptoms had a stronger influence on the mental component of HRQoL than on the physical component. This association was detected regardless of impairment in glucose regulation. We also found that non-melancholic depressive symptoms were associated with lower physical quality of life in all subjects regardless of glucose regulation status. Based on these findings, it seems that type of depressive symptoms does not modify the association between glucose regulation and HRQoL.

Previously known diabetes was associated with higher BDI scores, which is consistent with previous findings.^7 8 36^ No association was detected between pre-diabetes and newly diagnosed diabetes and BDI score. Other studies have also been unable to find an association between depression and pre-diabetes and newly diagnosed diabetes.^37 38^ It has previously been suggested that the burden of diabetes as a disease is responsible for the decline in mental health among people with diabetes, which could explain our findings as they indicate that pre-diabetes is not linked with depressive symptoms nor poorer mental functioning.^39^

We found that HRQoL was lower among subjects with previously known diabetes, but not among those with pre-diabetes and newly diagnosed diabetes. In line with our findings, it has been suggested that longer duration of diabetes, but not pre-diabetes, is related to decreased HRQoL.^4 5 16^ Decreased HRQoL related to years of living with diabetes may be attributed to higher prevalence of comorbidities and complications, such as neuropathy and retinopathy that have been associated with lower HRQoL.^40 41^ Unfortunately, we were unable to adjust for specific diabetes complications, which might affect our findings. The PCS was lower among subjects with previously known diabetes compared with the other subgroups. Despite this, previously known diabetes was not associated with a decrease in the physical role limitations domain nor with the bodily pain domain of the SF-36 score. Physical inactivity and obesity affect physical performance, and both are known risk factors for developing diabetes^42^; however, even after adjusting for them, there was a significant association between impaired glucose regulation and poorer physical functioning. According to our adjusted models, BMI strongly affects the associations between diabetes and poorer HRQoL.

As expected, both types of depression lowered MCS more than the PCS in all subgroups of glucose regulation. Subjects with symptoms of non-melancholic depression were associated with lower physical HRQoL than subjects with BDI score <10 or symptoms of melancholic depression. This association was independent of diabetes status, although most clearly seen in subjects with NGT and previously known diabetes. This is to the best of our knowledge the first study with the aim to investigate whether type of depressive symptoms affects HRQoL among the diabetic population. According to previous studies, non-melancholic depressive symptoms have been found to associate with elevated fasting glucose and IGT^13^ as well as with metabolic and inflammatory conditions,^12 14^ and has been suggested to originate from a systemic inflammation in the organism, whereas melancholic depression is thought to be purely a product of imbalances in the function of the central nervous system.^14 15 43^ This theory suggests that non-melancholic depression shares a common underlying denominator with other systemic diseases, such as diabetes, which our findings support.

Our study has several strengths. The cohort is well characterized, and our results were produced using both clinical and registered-based data. We were able to control for several factors known to influence both prevalence of depression and HRQoL. We used an OGTT to assess glucose regulation status, the validated SF-36 for assessing HRQoL, and the standardized BDI questionnaire for assessing depression. We used the summary scores of the SF-36 as well as the component scores. The study also has some limitations. Our study sample is from a homogeneous, restricted area in Finland and the findings from this study might therefore have to be cautiously implemented in other populations. As a result, the sample selected may not be representative of the entire Finnish population and the findings may not be generalized to represent the Finnish population. Although our sample is large when it comes to glucose regulation status, we may have limited power to investigate association related to the non-melancholic depressive symptoms. Due to the cross-sectional study design, we were unable to assess causal relationships. Lastly, although we did take in consideration comorbidities, we were unable to adjust for complications of diabetes.

In conclusion, both the non-melancholic and melancholic type of depressive symptoms affect mental HRQoL more severely than physical HRQoL regardless of impairment in glucose regulation. Symptoms of non-melancholic depression are associated with lower physical HRQoL in all subjects, especially in normoglycemic subjects and in subjects with previously known diabetes. Thus, our findings support the necessity of preventing pre-diabetes from turning into diabetes and identifying the type of depression to maintain high HRQoL. Resources should also be focused on preventing a decline in HRQoL and mental health in all stages of impaired glucose regulation. Further research is needed to investigate the association between diabetes and non-melancholic depressive symptoms.
